# Development of Serous Ovarian Cancer is Associated with the Expression of Homologous Recombination Pathway Proteins

**DOI:** 10.1007/s12253-014-9776-8

**Published:** 2014-04-22

**Authors:** Qingqing Ye, Li Chen, Xiaolu Yin, Yuan Jie Charles Liu, Qunsheng Ji, Enfeng Zhao

**Affiliations:** 1 Innovation Center China, AstraZeneca Global R&D, Shanghai, China; 2 Department of Obstetrics & Gynecology, Chinese PLA General Hospital, Beijing, China; 3 Department of Obstetrics & Gynecology, Hainan Branch of Chinese PLA General Hospital, 572014 Sanya, Hainan China

**Keywords:** Serous ovarian cancer (SOC), Homologous recombination (HR), Immunohistochemistry (IHC), Meiotic recombination 11 (Mre11), Mediator of DNA damage checkpoint protein 1 (MDC1), Ataxia telangiectasia mutated (ATM), ATM-Rad3-related (ATR), Breast cancer susceptibility gene 1 (BRCA1)

## Abstract

To investigate the expressions of key markers in the homologous recombination (HR) pathway and the correlation with clinicopathological parameters in serous ovarian cancer (SOC). We analyzed the protein expression of MRE11, MDC1, ATM, ATR and BRCA1 by immunohistochemistry (IHC) in 97 SOC samples, and correlated with clinical parameters including age, tumor grades, clinical stage, status of menstruation and chemotherapy. Low expression of MRE11 and MDC1 was detected in 14.4 % and 3.1 % of the patient samples, and negative expression of ATM, ATR and BRCA1 was found in 11.3 %, 6.3 % and 29.9 % of the patient samples, respectively. ATR deficiency was significantly associated with menopause (*P* = 0.025), strong expression of ATM (*P* = 0.017) and MRE11 (*P* = 0.040) with pre-menopausal SOC, strong expression of MRE11 (*P* = 0.016) with low tumor grade, and strong expression of BRCA1 (*P* = 0.015) with early clinical stage. In addition, low expression of MRE11 was strongly associated with negativity of ATR (*P* < 0.001) and BRCA1 (*P* = 0.004) Furthermore, ATR deficiency was associated with low expression of ATM (*P* = 0.028) and loss expression of BRCA1 (*P* = 0.009). Our results suggest that reduced expression or loss of proteins in HR pathway is associated with SOC development. Abnormality of MRE11 and BRCA1 are strongly associated with late clinical stage in SOC patients.

## Introduction

DNA double-strand breaks (DSBs) are particularly hazardous to the cell since they cause base pair mismatch,[1] which is strongly associated with cancer susceptibility. Three mechanisms exist to repair DSBs: non-homologous end joining (NHEJ), microhomology-mediated end joining (MMEJ), and homologous recombination (HR).[2] HR-mediated repair requires one homologous sequence to accurately repair breaks. In contrast, MMEJ requires a 5–25 base pair microhomologous sequence, whilst NHEJ can function to directly religate broken ends in the absence of a homologous template. Moreover, HR repairs DSBs in the late S and G2 phases of the cell cycle when sister chromatids are readily available,[3] as opposed to MMEJ which occurs in S phase and NHEJ in the G0/G1 and early S phases. Sister chromatids are ideal templates for repair as they provide identical copies of the same chromosome. Therefore, HR plays an important role in the fidelity of DNA replication,[4] which is vital to the integrity and stability of the genome.

During an HR repair process, DNA lesions are first identified and several key repair proteins are recruited. These include checkpoint mediator proteins like the MRN complex (MRE11-NBS1-RAD50) and MDC1. The MRN complex slows down crossover progression in mitosis [5] and MDC1 facilitates signal transmission to downstream proteins.[6, 7] Next, ATM and the RAD3-related ATR kinase, part of the Phosphatidyl-Inositol 3 Kinase-like protein Kinase (PIKK) family, cascade signals which arrest cell cycle progression [8] and thus allow DNA repair to occur. RAD51 (RAD50 forms MRE11-NSB1-RAD50 complex) also interacts with breast cancer susceptibility genes 1 and 2 (BRCA1/BRCA2). Finally, ATM and ATR then directly phosphorylate BRCA1 and BRCA2 to enable activation of DNA repair [9].

Many reports associate the risk of tumorgenesis with alterations in the HR pathway.[10–12] Aberrations in MDC1 and MRE11 have been strongly linked to breast carcinogenesis [13, 14] and also reported in other cancers.[15–17] Mutations and loss of ATM can contribute to lymphoid malignancies [18] and familial breast and ovarian cancers.[19, 20] Hypomorphic mutations of ATR have been linked to BC and OC development,[21] and mutation and loss of the BRCA1 gene is widely reported to increase the risk of breast and ovarian cancer.[22, 23]

Ovarian cancer (OC) is one of the most common hereditary cancers in women and results in more annual deaths than any other gynecological malignancy.[24] In recent years, reports have emerged suggesting that HR deficiency is strongly linked to the development of OC.[25, 26] 3.7 % deleterious and 4.8 % missense mutations of ATM have been reported in familial OC.[20] *ATR* mutations have been analyzed in familial breast cancer (BC) and OC, and 23 nucleotide substitution variants discovered.[27] Mutations in *BRCA1/2*, the two most widely studied genes in the HR pathway, have shown a strong linkage with OC in numerous reports.[28] The majority of the deaths associated with these mutations were from ovarian cancer of the serous histological type (SOC).[29] Moreover, SOC is often detected at an advanced stage, at which time it has already become highly lethal.[30] To better understand the alteration of protein expression in HR pathway, and to provide potential patient selection biomarker for HR inhibitor in SOC, we have analyzed the immunohistochemical expression of MRE11, MDC1, ATM, ATR and BRCA1 in 97 serous ovarian cancers and association with clinicopathological parameters of the patients.

## Materials and Methods

### Patients and Treatment

The current study was approved by the ethics committee of Chinese PLA General Hospital in Beijing. This study analyzed samples from 97 patients who underwent ovarian cancer complete resection at from Nov. 2005 to Nov. 2009. All 97 patients were diagnosed with serous ovarian cancer (SOC) by 2 qualified pathologists which accounts for more than 40 % of ovarian malignancies. The median patient age was 55 years old (range 35–77). Among the 97 cases, 56.7 % (55/97) of patients had received chemotherapy after surgery and 68.1 % (66/97) of patients had entered into menopause.

### Immunohistochemistry (IHC) Study

All tumor samples were collected immediately after surgery, fixed in 10 % buffered formalin and then embedded in paraffin. Four μm-thick tissue sections were cut for IHC study. The slides were baked at 56 ºC for 1 h, followed by de-paraffinized in xylene and rehydrated through a graded series of ethanol concentrations. Antigen retrieval was performed in a pressure cooker for 5 mins using Target Retrieval Solution (Dako). Endogenous peroxidase activity was blocked by Peroxidase Blocking Reagent (Dako) for 5 mins. Primary antibodies (ATM, Epitomics, 1:50; ATR, Santa-cruz Technology, 1:100; BRCA1, Merck, 1:100; MDC1, Sigma, 1:500; MRE11, Abcam, 1:200) were then applied to the specimen for 1 h at room temperature, followed by incubation with labeled polymer-HRP anti rabbit or anti mouse secondary antibody (Dako) for 30 mins at room temperature. Thorough rinsing with TBST was performed after incubation with each reagent. The slides were visualized using DAB substrate-chromagen (Dako) and washed with deionized water before counterstaining with haematoxylin (Sigma). The slides were then dehydrated through a graded series of ethanol concentrations, cleared in xylene and coverslipped in DPX mounting medium. Cases with positive staining of aforementioned 5 biomarkers in previous study were used as positive control. Isotype-matched immunoglobulin fraction instead of primary antibodies used in the experiment served as negative control.

### Interpretation of IHC

The intensity of the staining as well as the percentage of positive cells was recorded. Staining intensity was scored from 0, 1+, 2+ to 3+ following the criteria: 0, if absence of staining was observed; 1+, if >10 % of the tumor cells had weak staining; 2+, if >10 % tumor cells had moderate staining; and 3+, if >10 % tumor cells had strong staining. Tumors with 1+, 2+, and 3+ expression were interpreted as positive and tumors with no expression (0 score) were interpreted as negative.

### Statistical Analysis

Logistic regression was used to assess the association of homologous recombination deficiency (HRD) expression with clinicopathological parameters and *P* values were computed from log-likelihood ratio test. *P* values <0.05 were considered to be statistically significant. Pearson correlation coefficient method was used to assess the correlation of co-expression of HRD pathway genes. The data analysis was performed using R version 2.11.0 on Unix.

## Results

### Expression of ATM, ATR, MDC1, MRE11 and BRCA1 in SOC

**Figure 1:**
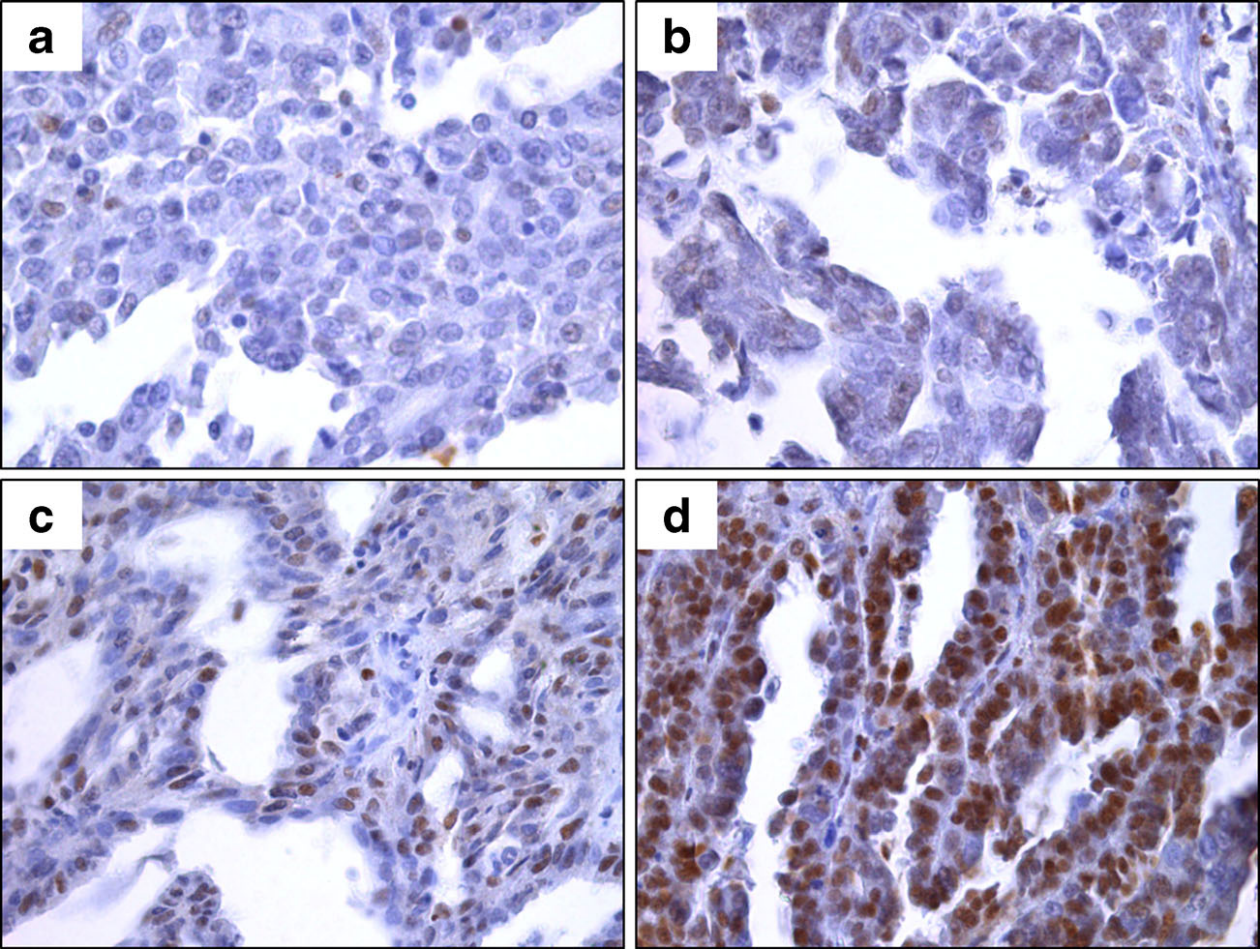
Representative images of ATM IHC staining with scoring 0, 1+, 2+ and 3+ in Chinese SOC. 1a-1d: negative, weak, moderate and strong staining of ATM

**Figure 2:**
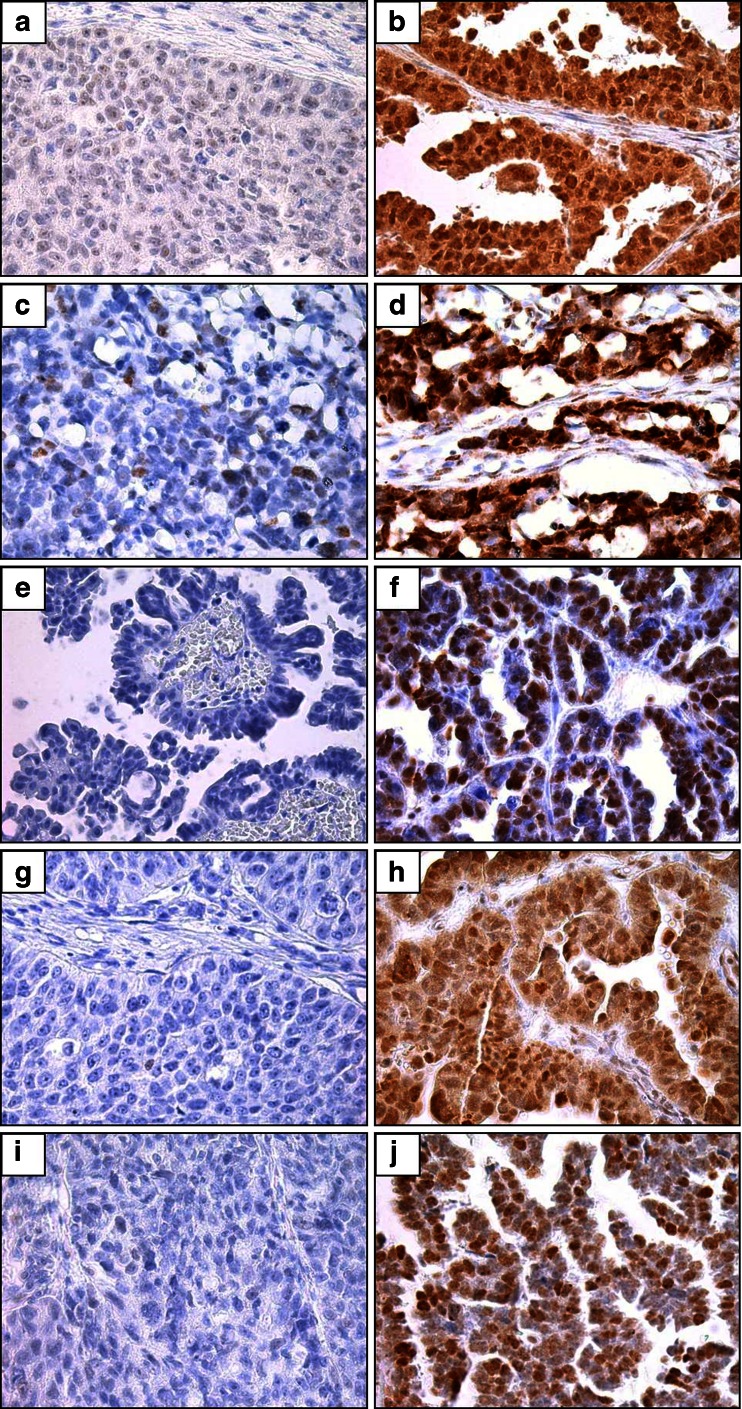
Representative images of IHC staining of MRE11, MDC1, ATM, ATR and BRCA1 in Chinese SOC. 2a-2b: Low and strong expression of MRE11; 2c-2d: Low and strong expression of MDC1; 2e-2f: negative and strong staining of ATM; 2G-2H: negative and strong staining of ATR; 2i-2j: negative and strong staining of BRCA1 in different SOC tumors, respectively. (Envision, 40 ×)

In our SOC samples, negative expression (IHC score ‘0’) of HR pathway proteins was at the following rates; ATM 11.3 % (11/97), ATR 6.3 % (6/96), and BRCA1 29.9 % (29/97). Low expression (IHC score ‘0’ and ‘1+’) of MRE11 was detected in 14.4 % (14/97) and MDC1 in 3.1 % of samples (3/97). (Figs. 1 and 2).

### Correlation Between Biomarkers Expression and Clinicopathological Parameters

Clinical information of the SOC patients recruited into this study was collected including age, tumor grade, clinical stage, status of menopause and chemotherapy. Statistical analysis of IHC data and clinicopathological parameters are shown in Table 1. Loss of expression of ATM, ATR and BRCA1 and low expression of MDC1 and MRE11 were not associated with age, clinical stage, tumor grade and chemotherapy status. However, loss of ATR expression was significantly correlated with menopause (*P* = 0.025). Although low or lost expression of these 5 proteins was not strongly associated with clinicopathological parameters, in-depth statistical analysis demonstrated that strong expression (IHC score ‘3+’) of MRE11 (*P* = 0.016) was significantly associated with low tumor grade (grade I and II). Furthermore, strong expression of ATM (*P* = 0.017) and MRE11 (*P* = 0.040), independently, was strongly associated with pre-menopausal SOC, and finally, strong expression of BRCA1 (*P* = 0.015) was significantly associated with early clinical stage (stage I and II). Detailed analysis results were shown in Table 2.

**Table 1:** Correlation study between HR marker’s expression and clinicopathological parameters in SOC patients (*log-likelihood ratio test*)

	MRE11 Expression (*n* = 97)		MDC1 Expression (*n* = 97)		ATM Expression (*n* = 97)		ATR Expression (*n* = 96)		BRCA1 Expression (*n* = 97)	
	MRE11-low expression/total cases %		MDC1-low expression/total cases %		ATM-negative/total cases %		ATR-negative/total cases %		BRCA1-negative/total cases %	
		*P*		*P*		*P*		*P*		*P*
Age (median)										
<55	4/47	0.517	1/47	0.827	6/47	0.507	0/47	0.300	15/47	0.577
	8.5 %		2.1 %		12.8 %		0.0 %		31.9 %	
> = 55	10/50		2/50		5/50		6/49		14/50	
	20.0 %		4.0 %		10.0 %		12.2 %		28.0 %	
Tumor grade										
I	1/2	0.174	0/2	0.092	2/2	0.212	1/2	0.826	1/2	0.913
	50.0 %		0.0 %		100.0 %		50.0 %		50.0 %	
II	2/34		0/34		4/34		1/34		10/34	
	5.9 %		0.0 %		5.9 %		2.9 %		29.4 %	
III	11/61		3/61		5/61		4/60		18/61	
	18.0 %		4.9 %		8.2 %		6.7 %		29.5 %	
Clinical stage										
I	0/4	0.542	0/4	0.343	1/4	0.844	0/4	0.174	1/4	0.578
	0.0 %		0.0 %		25.0 %		0.0 %		25.0 %	
II	1/9		0/9		1/9		0/9		2/9	
	11.1 %		0.0 %		11.1 %		0.0 %		22.2 %	
III	13/84		3/84		9/84		6/83		26/84	
	15.5 %		3.6 %		10.7 %		7.2 %		31.0 %	
Menopause										
Yes	12/66	0.184	3/66	0.120	8/66	0.143	6/65	0.025	18/66	0.375
	18.2 %		4.5 %		12.1 %		9.2 %		27.3 %	
No	2/31		0/31		3/31		0/31		11/31	
	6.5 %		0.0 %		9.7 %		0.0 %		35.5 %	
Chemotherapy										
Yes	8/55	0.911	3/55	0.063	5/55	0.864	3/54	0.745	16/55	0.969
	14.5 %		5.5 %		9.1 %		5.6 %		29.1 %	
No	6/42		0/42		6/42		3/42		13/42	
	14.3 %		0.0 %		14.3 %		7.1 %		31.0 %	

Low expression: IHC score 0 and 1+; Negative: IHC score 0

**Table 2:** Association analysis of clinicopathological parameters with MRE11, ATM and BRCA1 expression in SOC patients (*log-likelihood ratio test*)

Clinicopathological parameters		MRE11 Expression (*n* = 97)			ATM Expression (*n* = 97)			BRCA1 Expression (*n* = 97)	
	negative to moderate staining	strong staining	*P*-value	negative to moderate staining	strong staining	*P*-value	negative to moderate staining	strong staining	*P*-value
Tumor grade									
I/II	6	30	0.02	18	18	0.48	31	5	0.27
III	24	37		26	35		47	14	
Clinical stage									
I/II	2	11	0.21	5	8	0.54	13	0	0.01
III	26	56		39	43		64	18	
Menopause									
No	5	26	0.04	9	22	0.02	28	3	0.09
Yes	23	41		35	29		49	15	
Chemotherapy									
No	13	28	0.68	18	23	0.68	34	7	0.68
Yes	15	39		26	28		43	11	

Negative staining: IHC score 0; Weak staining: IHC score 1+; Moderate staining: IHC score 2+; Strong staining: IHC score 3+

Overall, 53/96 (55.2 %) SOC cases were identified with negative IHC expression of at least 1 HR pathway protein. Combined biomarker analysis results were shown in Table 3, which indicated that low expression (IHC score 0 and 1+) of MRE11 was strongly associated with loss (IHC score 0) of ATR (*P* < 0.001) and BRCA1 (*P* = 0.004). In addition, deficiency of ATR (IHC score 0) was strongly associated with low levels (IHC score 0 and 1+) of ATM (*P* = 0.028) and loss of expression (IHC score 0) of BRCA1 (*P* = 0.009). Expression of MDC1 did not show any association with expression of the other 4 HR pathway proteins.

**Table 3:** Co-expression analysis amongst individual HR biomarker expression in SOC patients

*P*-value	Mre11 low expression	MDC1 low expression	ATM low expression	ATR negative	BRCA1 negative
Mre11 low expression	/	0.3768	0.7425	0.0002	0.0044
MDC1 low expression	0.3768	/	0.5722	0.1778	1.0000
ATM low expression	0.7425	0.5722	/	0.0279	1.0000
ATR negative	0.0002	0.1778	0.0279	/	0.0091
BRCA1 negative	0.0044	1.0000	1.0000	0.0091	/

Low expression: IHC score 0 and 1+; Negative: IHC score 0

## Discussion

Ovarian cancer is the leading cause of death in gynecological malignancies. Although platinum-based chemotherapy regimens are widely used to treat OC patients, these are non-specific treatment modalities which can lead to severe side effects and poor tolerance due to effects on normal tissues. Recently, several reports have shown that deficiencies in the levels of key HR pathway proteins are associated with OC. However, this association has not been fully understood. To explore this further in a Chinese population, we measured the expression of key HR pathway proteins (MRE11, MDC1, ATR, ATM, and BRCA1) and analyzed this data for associations with clinicopathological parameters in 97 Chinese SOC patients.

MRE11 and MDC1 genetic abnormalities can contribute to cancer susceptibility.[15–17] Our data shows that the incidence of low expression of MDC1 and MRE11 was 3.1 % and 14.4 %, respectively. After statistical analysis, we found strong expression of MRE11 (*P* = 0.016) to be significantly associated with low tumor grade. Conversely, low levels of MRE11 appear to be associated with SOC differentiation. Previous reports have indicated genetic MRE11 abnormalities to be linked to development of various cancers, but protein levels have rarely been studied in SOC. To our knowledge, this study is the first to report an association of MRE11 protein expression with SOC development. Although MDC1 expression failed to show significant correlations with clinical stage and menopause, a strong trend was observed between tumor grade (*P* = 0.09) and chemotherapy treatment status (*P* = 0.06), warranting further investigations of this protein as a potential SOC prognostic biomarker.

ATM and ATR are important signaling kinases that activate a complex network of DNA damage response pathways. These coordinate cell cycle checkpoint and DNA repair functions.[8] Deficiencies in ATM and ATR can result in DNA damage sensitivity and cancer predisposition.[10–12] In the current study, loss of expression of ATM and ATR were detected in 11.3 % and 6.3 % of patient samples, respectively. Moreover, statistical analysis showed that loss of expression of ATR (*P* = 0.025) was significantly correlated with menopause, whilst strong expression of ATM (*P* = 0.017) correlated with tumors derived from pre-menopausal women.

BRCA1 and BRCA2 protein complexes play critical roles in halting cell cycle progression and enabling DNA repair. Mutations in *BRCA1* and *BRCA2*, which have been reported in various studies, account for 70-80 % and 15 % of families with a history of OC, respectively.[31, 32] Women with a familial history of OC are more likely to develop a deleterious mutation in *BRCA1*/*2*.[28] *BRCA1* mutations typically confer a higher risk of BC and OC than *BRCA2* mutations. Besides OC, deleterious *BRCA1* mutations may also increase a woman’s risk of developing cervical, uterine, pancreatic, and colon cancer.[33, 34] A recent study suggested that promoter hypermethylations, somatic mutations, and genomic deletions of *BRCA1* might be responsible for the loss or reduced expression of BRCA1 protein.[35] Here, we analyzed BRCA1 protein expression in SOC patients and found a negativity rate of 29.9 % (29/97). Strong expression was significantly correlated with early clinical stage (*P* = 0.015). Thus, our data indicates that loss of BRCA1 protein is associated with serous ovarian cancer progression.

In the co-expression analysis of all 5 proteins, we found the following associations: 1) low expression of MRE11 with loss of ATR (*P* < 0.001) and BRCA1 (*P* = 0.004) and 2) deficiency of ATR with low levels of ATM (*P* = 0.028) and expression loss of BRCA1 (*P* = 0.009). These results showed that reduced expression of MRE11 can influence the downstream expression levels of ATR and ATM. Furthermore, the expression level of BRCA1, a direct phosphorylation target of ATM, was significantly lower in patients with ATM loss.

To conclude, by profiling key HR pathway proteins in SOC patients we have demonstrated that protein expression changes of MRE11 and BRCA1 are strongly associated with serous ovarian cancer in late clinical stage, suggesting their potential utility as prognostic tools in the analysis of tumor biopsies or circulating tumor cells. Moreover, as SOC represents an especially lethal cancer with limited therapeutic options, we believe that these association studies further underpin the HR pathway as a novel area of potential therapeutic intervention for SOC.
